# Artemether-Soluplus Hot-Melt Extrudate Solid Dispersion Systems for Solubility and Dissolution Rate Enhancement with Amorphous State Characteristics

**DOI:** 10.1155/2013/151432

**Published:** 2013-04-04

**Authors:** Ritesh A. Fule, Tarique S. Meer, Ajay R. Sav, Purnima D. Amin

**Affiliations:** Department of Pharmaceutical Sciences and Technology, Institute of Chemical Technology, Nathalal Parekh Marg, Matunga, Mumbai 400019, India

## Abstract

This work studied artemether (ARTM) solid dispersion (SD) formulation using mixture of polymer excipient Soluplus, PEG 400, Lutrol F127, and Lutrol F68 melts at temperatures lower than the melting point of ARTM using a laboratory-size, single-screw rotating batch extruder. The effects of three surfactants PEG 400, Lutrol F127, and Lutrol F68 and parameters like mixing temperature, screw rotating speed, and residence time were systematically studied. SEM, XRD, and FT-IR were employed to investigate the evolution of ARTM's dissolution into the molten excipient. Differential scanning calorimetry (DSC) was used to quantitatively study the melting enthalpy evolution of the drug. The results showed that the dissolution rate increased with increasing the ratio of polymer and surfactant to that of drug. It was concluded that the dissolution of the drug in the polymer melt is a convective diffusion process and that laminar distributive mixing can significantly enhance the dissolution rate. The aqueous solubility and dissolution rate of prepared solid dispersion were significantly enhanced. In vitro antimalarial studies revealed marked improvement in IC_50_ values. Thus hot-melt extrusion (HME) is a promising technology for improving solubility and dissolution profile of ARTM.

## 1. Introduction

In this study, a solid dispersion approach using hot-melt extrusion (HME) was deliberated to improve the dissolution rate of a poorly water-soluble and low glass transition temperature (*T*_*g*_) drug ARTM. The most cited methods in the literature to formulate solid dispersions are melting of excipients or fusion method, embedding of drug by means of spray drying [1], coevaporation [2], coprecipitation [3], freeze-drying, and roll-mixing or comilling [4]. For the purpose of this discussion, the drug/polymer system can be defined as solid dispersion when the drug is dissolved at a molecular level; that is, when the drug forms one phase system with polymer. The mixtures of drug and polymer should show single glass transition temperature or drug should be present in amorphous state are the ideal parameters for prepared solid dispersion [5]. The amorphous solids have particles dispersed molecularly inside structure of a liquid. The addition of a high-*T*_*g*_ (glass transition temperature) polymer elevates the *T*_*g*_ of the amorphous system. Thermodynamically the drug has a lower chemical potential when mixed with a polymer, resulting in the change of crystallization driving force. It is also generally accepted that drug-polymer intermolecular interactions are important for the stabilization of the solid dispersion [5]. Crowley et al. have published extensive information regarding the factors affecting stabilization of amorphous state [6]. The critical factors affecting stability of amorphous state of solid dispersion are the glass transition temperature (*T*_*g*_), hygroscopic nature, purity, and storage conditions. Processing of solid dispersions below the active ingredient melting point has also been well established as a method to produce amorphous solid dispersions [7]. The *T*_*g*_ of the drug can be increased by adding polymers with high *T*_*g*_ values. In drug/polymer system, the stability of the amorphous form primarily depends on criteria such as drug and polymer interaction, viscosity of polymer, and glass transition temperature of the mixture [8]. The literature has shown that higher glass transition temperature and higher viscosity of polymers usually show superior stability for the amorphous drug [9]. The specific interactions between drug and polymer are important considerations for stabilization of the amorphous formulation [10]. Therefore the evaluation and selection of polymer are key factors in developing solid dispersions. For this study the hot-melt extrusion technology was utilized to prepare solid dispersion of the poorly water-soluble model drug ARTM. This technology employs application of high shear and high temperature to formulate solid dispersions. This technology has many advantages over traditional processing techniques such as spray drying or coevaporation which involves organic solvents [11]. Most important advantages are solvent-free continuous process and relatively feasible scale-up.

ARTM is a poorly soluble and poorly permeable BCS class IV drug used for prevention of malaria. ARTM is a potent antimalarial agent accessible for the treatment of severe multiresistant malaria and is included in WHO list of essential medicines (WHO web site). It is active against *P. vivax* as well as chloroquine-sensitive and chloroquine-resistant strains of *P. falciparum* and is also indicated in the treatment of cerebral malaria. However, the therapeutic potential of ARTM is substantially delayed due to its low oral bioavailability (*~*40%). The low bioavailability of ARTM shoots from its poor aqueous solubility [12].

In the present study polyvinyl caprolactam-polyvinyl acetate-polyethylene glycol graft copolymer (Soluplus), a new polymer with amphiphilic properties, was used. Soluplus shows exceptional solubilizing properties for BCS class II and class IV drugs and also offers the possibility of producing solid dispersions by hot-melt extrusion [13]. The dissolution of poorly soluble drugs in aqueous media can be highly improved by the use of solid dispersions with Soluplus [14]. ARTM was selected as the poorly water-soluble drug and Soluplus, Lutrol F127, Lutrol F68, and PEG 400 were selected as hydrophilic polymers. Lutrol F grades are white coarse grinded powders with waxy consistency with good solubilizing properties for solid dispersion formulations. Lutrol F127 (Poloxamer) has melting point 52°C and higher molecular weight than Lutrol F68. Lutrol F68 (Poloxamer) has melting point 56°C and less molecular weight than Lutrol F127 [15].

The primary objective of this study was to obtain stable solid dispersion of poorly water soluble and low *T*_*g*_ model drug ARTM with water soluble/ionic polymer and permeability enhancer polymers. The secondary objective was to evaluate performance attributes of solid dispersion as a function of polymer-type and concentrations.

## 2. Materials and Methods

### 2.1. Materials

Artemether was generous gift by Bajaj healthcare pvt. Ltd. India. The Soluplus, Lutrol F127, and Lutrol F68 were provided by BASF (Ludwigshafen, Germany). PEG 400 of analytical grade was procured from sd. Fine chemicals, Mumbai, India. All other chemicals used were of analytical grade or equivalent quality.

### 2.2. Formulation and Processing Techniques to Prepare Solid Dispersions

#### 2.2.1. Preparation of Melt Extrudates

Solid dispersions (SDs) were prepared by hot-melt extrusion in a single-screw extruder (Manufactured by S.B. Panchal ltd, Mumbai, India). Extrusion parameters were adjusted for drug and polymers are summarized in Table 1. Die used for extrusion was of 2 mm diameter. ARTM was mixed with Soluplus at drug/polymer mass ratios of 1 : 4, 1 : 5, 1 : 6, 1 : 7, 1 : 8, and 1 : 9 using a mortar and pestle for 5 min. The prepared physical mixtures (PMs) were extruded using a corotating single-screw extruder at a screw speed of 50 rpm. ARTM + Soluplus mixtures were extruded at a temperature of 84°C. ARTM + Soluplus + PEG 400 physical mixtures containing 1 : 1.8 : 0.2, 1 : 2.8 : 0.2, and 1 : 3.8 : 0.2 ratios were extruded at a temperature of 82°C, whereas ARTM + Soluplus + Lutrol F127 in ratio 1 : 1.8 : 0.2, 1 : 2.8 : 0.2, and 1 : 3.8 : 0.2 were extruded at 80°C. ARTM + Soluplus + Lutrol F68 physical mixtures containing 1 : 1.8 : 0.2, 1 : 2.8 : 0.2, and 1 : 3.8 : 0.2 ratios were extruded at temperature of 77°C. The melt extrudates were grinded using grinder, passed through a 200 *μ*m sieve, transferred to glass vials with stopper, and stored in a desiccator. A suitable quantity of the physical mixture and solid dispersion from each drug loading was kept for analysis. However, to date, the effect of this processing method on polymer stability has not been explored. Processing speed and ejection temperature were identified as controllable parameters in the process that could potentially affect polymer molecular weight or chemical stability. A 2-level factorial experimental design, presented in, was implemented to investigate effect of these parameters on polymer degradation.

#### 2.2.2. Analysis of Drug Content

The SD equivalents to 20 mg of ARTM were dissolved separately in 50 mL of phosphate buffer (pH 7.2). The solution was filtered and further diluted so that the absorbance fell within the range of standard curve. The samples were filtered through a 0.45 mm membrane filter and the drug content was determined spectrophotometrically at 211 nm as shown in Figure 3. The blank formulation was treated in the same manner as the ARTM formulation.

### 2.3. Preliminary Solubility Studies

#### 2.3.1. Saturation Solubility Study

**Figure 1:**
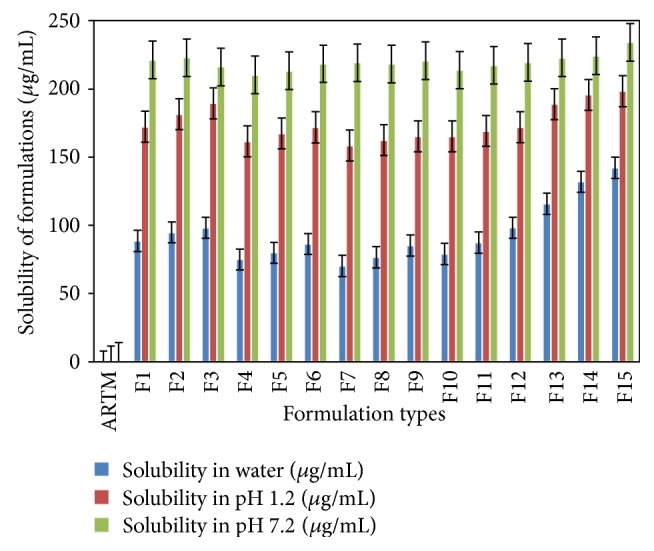
Solubility of ARTM and its SD formulations in water, pH 1.2 and pH 7.2 (mean ± SD (*n* = 3)).

An excess quantity of ARTM was placed in 20 mL capacity test tubes containing 10 mL of different solutions (distilled water, 0.1 N HCl and phosphate buffer at pH 7.2) separately. The samples were sonicated for 20 min at room temperature and capped glass test tubes were shaken for 48 h at 37 ± 0.1°C, speed 75 rpm using orbital shaking thermo stable incubator (Boekel Scientific, Germany). The solutions in the test tubes were kept for centrifugation for 20 min at 10000 rpm. The supernatant solution was then passed through a whatman Filter Paper (Grade 1) and the amount of the drug dissolved was analyzed spectrophotometrically (UV-1601PC, Shimadzu, Japan) at 211 nm for ARTM. All solubility measurements were performed in triplicate as shown in Figure 1.

#### 2.3.2. Phase Solubility Study [16]

**Figure 2:**
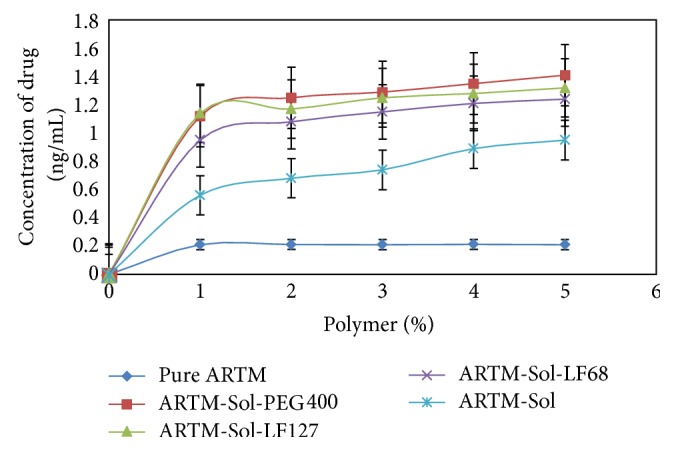
Phase solubility of drug ARTM and HME SD formulations in water (mean ± SD (*n* = 3)).

**Figure 3:**
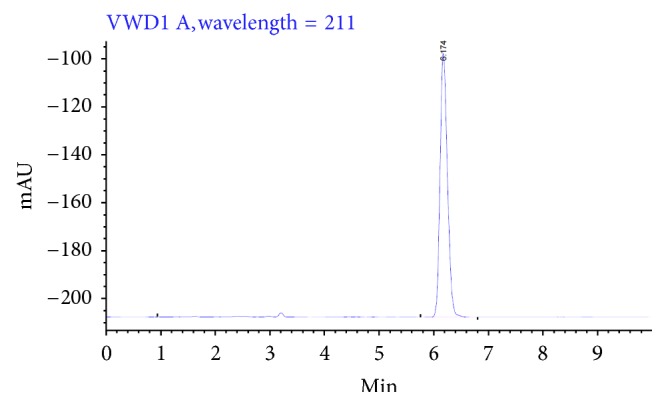
HPLC chromatogram of pure ARTM.

Phase solubility study was performed according to the method described by Higuchi and Connors. An excess amount of ARTM was placed in 20 mL test tubes containing in 10 mL of distilled water with different concentrations of Soluplus separately. Soluplus (1%, 2%, 3%, 4%, and 5% w/v) was used as hydrophilic polymer. In the formulation containing mixture of ARTM + Soluplus + PEG 400 the equivalent% of PEG 400 (i.e., ratio of surfactants used in different SD formulations) was also added along with Soluplus (1%, 2%, 3%, 4%, and 5% w/v). In the formulation containing mixture of ARTM + Soluplus + Lutrol F127 and ARTM + Soluplus + Lutrol F68 the equivalent% of Lutrol F127 and Lutrol F68 (i.e., ratio of surfactants used in different SD formulations) was also added, respectively, along with Soluplus (1%, 2%, 3%, 4%, and 5% w/v). Test tubes were covered with cellophane membrane to avoid solution loss and then shaken (75 agitations/min) in orbital shaking incubator (Boekel Scientific, Germany) for 48 h at 37°C. The solutions in the test tubes were kept for centrifugation for 20 min at 10000 rpm. 5 mL of supernatant was withdrawn and filtered through Whatman Filter Paper (Grade 1). The filtrates were analyzed using a UV-visible spectrophotometer at 211 nm after suitable dilution. All solubility measurements were performed in triplicate Figure 2.

#### 2.3.3. Gibbs-Free Energy (Δ*G*°tr) Calculation [17]

The Δ*G*°tr value provides information about whether the treatment is favourable or unfavourable for drug solubilization in an aqueous medium. Negative Gibbs-free energy values indicate improved dissolution. The Δ*G*°tr values of ARTM were calculated using the following equation:

(1) $$\begin{array}{c} \Delta G^{{}^{\circ}}\mathrm{tr}=\left\{-2.303RT\text{Log}\left(\frac{S_{0}}{S_{s}}\right)\right\}, \end{array}$$

where *S*_0_/*S*_*s*_ is the ratio of the molar solubility of ARTM before and after treatment with mixture of polymer Soluplus and surfactants. The value of gas constant (*R*) is 8.31 J K^−1^ mol^−1^ and  *T*  is temperature in degree kelvin. The order of phase solubility and Δ*G*°tr of ARTM at different formulations containing Soluplus, PEG 400, Lutrol F127, and Lutrol F68 are shown in (Tables 3, 4, 5, and 6). Negative values of Gibbs-free energy indicate improved dissolution.

#### 2.3.4. Stability Indicating HPLC Method Development [18]

The assay of the SD was evaluated using high-performance liquid chromatography (HPLC) apparatus equipped with Binary HPLC pump and 2998 Photodiode Array detector (Agilent Corporation, Milford, USA). A reverse-phase C18 column (150 × 4.6 mm; 5 *μ*m particles) was used. The mobile phase was composed of water acetonitrile (25 : 75, v/v). Samples equivalent to 20 mg of ARTM were dissolved in 5 mL of methanol and appropriately diluted and the drug content was determined by HPLC at  *λ* = 211 nm. The method developed was found to be stable for acid, base, oxidation, reduction, and heat degradation studies. Flow rate and injection volume were 1 mL/min and 20 *μ*/L for. Inter- and intraday coefficients of variation for ARTM were found to be ≤10% [19].

#### 2.3.5. Moisture Uptake and Stability Studies [20]

A weighed amount of prepared SD about 100 mg was placed in crucibles at accelerated condition of temperature and humidity, 40 ± 2°C and 75 ± 5% RH, respectively, in environmental test chamber (Thermo lab, India). The changes in weight of samples were determined using Moisture balance MB 50C (CITIZEN, India).

#### 2.3.6. Flowability of SD [21]

The flowability of prepared SD was characterized by measuring angle of repose and Carr's compressibility index. Angle of repose was determined by pouring the dispersion powder through a funnel (10 mm diameter orifice) onto a flat surface and measuring the angle between the horizontal and the slope of the heap of granules. Bulk density was calculated by measuring the volume of 5 g powder in a 10 mL cylinder. The cylinder was tapped 100 times until no further reduction in the volume of the SD powder was observed. Tapped density was calculated using the volume of the SD powder after tapping.

## 3. Solid State Characterisation

### 3.1. Differential Scanning Calorimetry

Differential scanning calorimeter (DSC-PYRIS-1, Perkin Elmer, USA) was used to study the drug polymer interactions and thermal behavior of drug. The experiments were performed in a dry nitrogen atmosphere. The samples were heated at a rate of 10°C min^−1^ from ambient temperature to the melting point. Samples (5.0–10.0 mg) of ARTM, Soluplus, Lutrol F127, and Lutrol F68 and extrudates were accurately weighed into crimped aluminium pans and heated at 10°C/min under a nitrogen purge (20 mL/min) from 0°C to 120°C. Physically mixed samples were cooled rapidly from 120°C/min to 40°C and reheated at 10°C/min (second heating cycle) to 120°C. In order to understand the miscibility of Soluplus and ARTM, SDs were investigated. An empty crimped aluminium pan was used as the reference cell. The DSC was calibrated for baseline using empty cells and for temperature.

### 3.2. Flory-Huggins Modelling [22]

The Flory-Huggins (FH) interaction parameter (*χ*) was estimated from melting point, and depression data was calculated using the following equation:

(2) 1Tmmix−1Tmpure  =−RΔHf(lnΦdrug+(1−1m)Φpolymer   −RΔHfcc+ χΦ2polymer(1−1m)),

where *T*_*m*_mix  is the melting temperature of the drug in the presence of the polymer, *T*_*m*_pure  is the melting temperature of the drug in the absence of the polymer, Δ*H*_*f*_ is the heat of fusion of the pure drug, *m* is the ratio of the volume of the polymer to that of ARTM, and  Φ_drug_  and  Φ_polymer_  are the volume fractions of the drug and the polymer, respectively.

### 3.3. Powder X-ray Diffractometry

Powder X-ray diffraction patterns were collected using a Miniflex apparatus (Rigaku, Japan) with CuK*α*  radiation. Samples were placed on a zero background sample holder and incorporated on a spinner stage. Cu-K′′1 radiation was used as X-ray source. Soller slits (0.04 rad) were used for the incident and diffracted beam path. The results were recorded over a range of 0–40° (2*θ*) using the Cu-target X-ray tube and Xe-filled detector. The operating conditions were voltage 40 kV, current 20 mA, scanning speed 1/min, temperature of acquisition: room temperature, detector: scintillation counter detector, and sample holder: nonrotating holder.

### 3.4. FTIR Spectroscopy

Fourier transform infrared analysis was performed on samples of crystalline and amorphous ARTM, Soluplus melt extrudates, and PMs of drug using a Fourier transform infrared spectrophotometer model 4100 (Spectrum GX-FT-IR, Perkin Elmer, USA). Samples were mixed with dry potassium bromide using a mortar and pestle, compressed to prepare a disk, and analysed over a range 4000–400 cm^−1^.

### 3.5. Scanning Electron Microscopy (SEM)

The surface characteristics of samples were studied by scanning electron microscopy (SEM). Double-sided carbon tape was affixed on aluminum stubs. The powder sample was sprinkled onto the tape. The aluminum stubs were coated with platinum plasma beam using JFC-1600 auto fine coater to make layer of 2 nm thickness above the sprinkled powder for 25 minutes. Then, these stubs were placed in the vacuum chamber of a scanning electron microscope. The samples were observed for morphological characterization using a gaseous secondary electron detector (working pressure: 0.8 Torr, acceleration voltage: 30.00 kV) XL 30. Model JEOL 5400 made in Japan was used during analysis.

## 4. In Vitro Dissolution Testing

### 4.1. Dissolution Studies

The in vitro drug dissolution properties were examined according to the International Pharmacopeia (IP) basket method (IP 2009). Samples equivalent to 20 mg of ARTM containing SD filled inside capsules were added to 1000 mL phosphate buffer of pH 7.2 with 1% SLS (sodium lauryl sulphate) at a temperature of 37 ± 0.2°C. The solution was stirred with a rotating basket at 100 rpm. Samples (5 mL) were withdrawn from each vessel at predetermined time intervals (10, 20, 30, 40, 50, 60, and 120 min), filtered over a cellulose acetate filter of 0.45 *μ*. At each time point, the same volume of fresh medium was replaced. The concentration of ARTM in each sampled aliquot was determined using an ultraviolet (UV) visible spectrophotometer at 211 nm and a standard calibration curve that was linear over the UV absorbance range.

### 4.2. Stability Study

Stability studies were conducted by placing powdered samples in stoppered glass vials which were stored in a controlled temperature environment inside stability chamber with relative humidity (RH) of 75% and 40°C temperature. Samples were removed after 6 months and tested for crystalline content using DSC and XRD. Drug release experiments were also conducted on samples stored for 6 months and compared with those tested immediately following preparation. The assay of the SD was evaluated using high-performance liquid chromatography (HPLC) and the mobile phase was composed of water acetonitrile (25 : 75, v/v). Samples equivalent to 20 mg of ARTM were dissolved in 5 mL of methanol and appropriately diluted, and the drug content was determined by HPLC at  *λ* = 211 nm.

### 4.3. Dissolution Kinetic Studies

Dissolution kinetic studies of prepared formulation were carried out using zero order, first order, Higuchi, Hixson Crowell, and korsemeyer Peppas equation model. Regression coefficient factor (*r* ^2^) and other factors were calculated to understand the release kinetic behaviour of prepared SD formulations.

### 4.4. Statistical Analysis

The effect of formulation on drug solubility/dissolution properties was statistically analyzed using a repeated measures one-way ANOVA. Individual differences in drug dissolution between formulations were statistically identified using Fisher's PLSD test. In all cases *P* < 0.05 denoted significance.

## 5. Antimalarial Drug Screening Assay

The compounds were tested for in vitro antimalarial activity against *Plasmodium falciparum *3D7 (chloroquine-sensitive cell lines), ITG (chloroquine-resistant cell lines) using the SYBR Green-I staining technique, as described earlier [23].

### 5.1. SYBR Green Assay of Plasmodium Viability

Lysis Buffer was made by adding Tris HCl (20 mM; pH 7.5), EDTA (5 mM), Saponin (0.008% w/v), and Triton X-100 (0.08% v/v).

### 5.2. Standardization

*Plasmodium falciparum *culture was serially diluted with nonparasitized erythrocytes and medium to yield a haematocrit of 1% and parasitemia levels ranging from 0 to 12% to obtain a standard curve. Then a volume of 100 *μ*L of the serially diluted culture was dispensed into a 96-well plate in triplicates, immediately followed by the addition of 100 *μ*L of SYBR Green I in lysis buffer (0.2 *μ*L of SYBR Green I/mL of lysis buffer). The plate was wrapped in aluminum foil and incubated on a shaker at RT for 30–60 minutes. The fluorescence was measured at 458 and 541 nm. The background fluorescence was subtracted for the empty well, and the nonparasitized erythrocytes were analyzed by linear regression. A similar procedure was followed for the test samples. The 96-well microplate was read using the HTS 7000 plus, bioassay reader (Perkin Elmer). The IC_50_ value was expressed as the drug concentration and various HME formulations (F1 to F15) resulting in a 50% inhibition of number of schizonts with three or more nuclei per 200 parasites by comparison with the drug-free control. The IC_50_ values for both methods were calculated by nonlinear regression analysis. The threshold IC_50_ for in vitro resistance to ARTM was defined as between 1 to 100 nM.

## 6. Results and Discussion

Initially lower drug to Soluplus ratio (1 : 1, 1 : 2, 1 : 3) was taken and extruded to prepare solid dispersions. But the extrudes obtained were sticky, not uniform, and unable to get powdered. Henceforth, we perform the process with higher ratios and implement the use of various surfactants for the ease of process as well as improve uniformity. Higher ratios showed good extrudability with uniformity. Surfactant was used in very small concentration which is studied during optimization. Here, we are comparing the effect of Soluplus in the presence and absence of surfactants.

Solid-dispersion approaches to drug dissolution enhancement typically involve the generation of a solid solution in which the drug is present in a metastable amorphous state possessing a high internal energy and specific volume. Consequently in the formulation and design of solid dispersions it becomes extremely important to have analytical methods that allow for screening of these factors and the role they may have on the physicochemical properties of the dispersion. In particular, methods such as DSC, PXRD, drug dissolution, SEM, and spectroscopic FTIR techniques are often used for current research studies.

### 6.1. Solubility Studies

The solubility of the drug in the presence of concentrated solutions of a polymeric carrier can help determine the mechanism of dissolution from a solid dispersion. To examine the solubilizing power of Soluplus and used surfactants, the equilibrium solubility of crystalline ARTM in phosphate buffer of pH 7.2 containing polymer was determined and compared to the equilibrium solubility in buffer 7.2 in the absence of polymer. Aqueous solubility of all the formulations was carried out and compared with that of pure ARTM. Study revealed that the solubility of ARTM as well as formulation mixtures are found to be higher in buffer of pH 7.2 than that of water, which can be used as dissolution medium in further analysis.

### 6.2. Theories and Calculation

#### 6.2.1. Gibbs-Free Energy Calculation

The Δ*G*°tr value provides information about whether the treatment is favorable or unfavorable for drug solubilization in an aqueous medium. Negative Gibbs-free energy values indicate improved dissolution.

#### 6.2.2. Flory-Huggins Modelling

Flory-Huggins lattice theory was developed to better understand the thermodynamics of polymer-drug mixtures by taking into account the entropy of mixing of a drug molecule with the polymer, as well as any enthalpy of mixing contributions. Numerous workers have applied various forms of the FH equation to better understand pharmaceutical systems, including solid dispersion systems. It has been reported that if coexistence  *χ* ≥ 0.5/*M*, so there is a presence of slightest degree of unfavorable non-bonding interactions between the drug, polymer, and surfactant mixture which may increase the stability even in amorphous state. As shown in Table 7 the value of FH interaction factor is (*χ*) not more than or equal to  0.5/*M*. This is because the entropy of mixing is greatly reduced due to formation of molecular dispersion using HME which means that it is favorable for mixing drug and polymer. Adhesive interaction between drug and polymer is favoured by the reduction in the *T*_*g*_ of SD systems which implicates the miscibility of drug and polymer. It indicates that the developed solid dispersions are thermodynamically stable. The contour surface plot of various parameters used in Flory-Huggins modelling was obtained as shown in Figure 18. The left-hand side parameter of equation, that is, 1/*T*_*m*_mix and 1/*T*_*m*_pure  were treated as independent variables and all the other parameters on right-hand side of equation are treated as dependent variables.

### 6.3. Solid State Characterisation

#### 6.3.1. DSC Analysis

**Figure 4:**
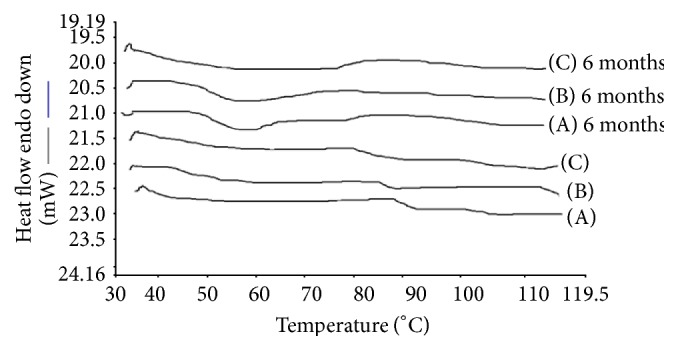
DSC thermograms of HME formulations: (A) ARTM-Sol-PEG 400 (1 : 2), (B) ARTM-Sol-Lutrol F127 (1 : 2), and (C) ARTM-Sol-Lutrol F68 (1 : 2).

**Figure 5:**
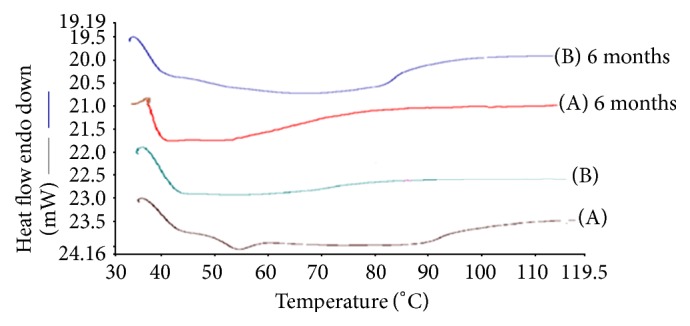
DSC of HME formulations: (A) ARTM-Sol (1 : 4), (B) ARTM-Sol (1 : 9).

**Figure 6:**
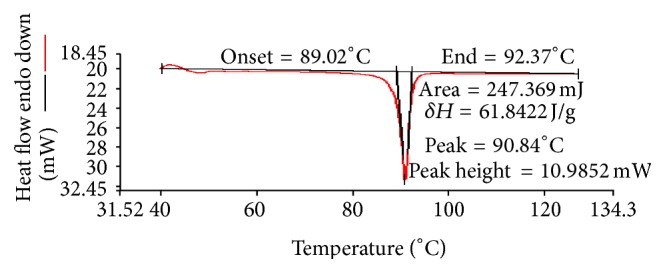
DSC thermogram of pure ARTM.

The DSC thermograms show that the crystalline ARTM was characterized by a single, sharp melting endotherm at 90°C (Δ*H*  61.842 Jg^−1^). The melting endotherm of the ARTM in the physical mixture occurred at 81°C, whereas the melt extrudates had no distinct melting endotherm for the drug. The formation of amorphous solid dispersion is attributed to the molecular interaction between drug and polymer. This indicated that the drug exists in the amorphous state in the melt extrudates. The disappearance of the melting endotherm in the DSC scan of HME suggested that the drug has been converted to the amorphous form during the extrusion process (Figures 4, 5, and 6).

#### 6.3.2. XRD Analysis

**Figure 7:**
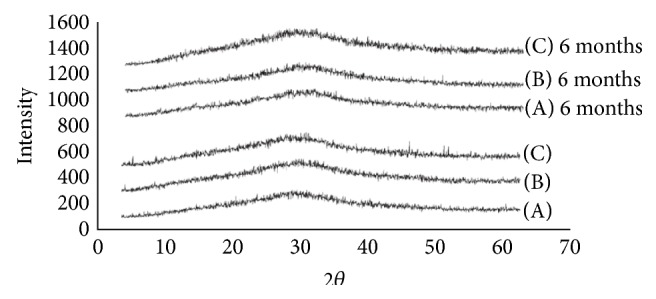
XRD patterns of (A) ARTM-Sol-PEG 400 (1 : 2), (B) ARTM-Sol-Lutrol F127 (1 : 2), and (C) ARTM-Sol-Lutrol F68 (1 : 2).

**Figure 8:**
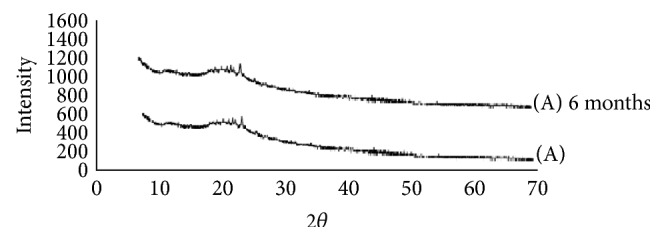
XRD pattern of (A) ARTM Sol (1 : 4).

**Figure 9:**
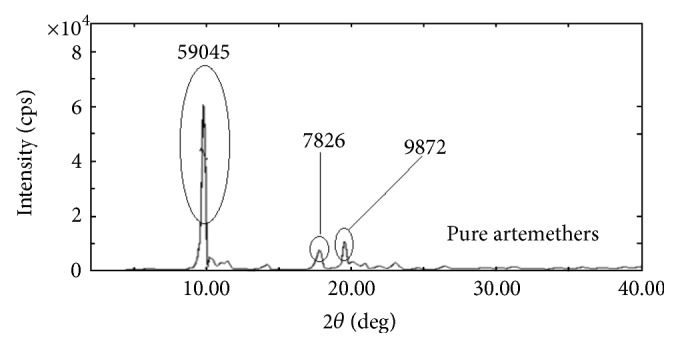
XRD pattern of pure ARTM.

The X-ray diffractograms of ARTM show sharp multiple peaks, indicating the crystalline nature of the drug. Several distinct peaks similar to crystalline ARTM were observed in the physical mixture of polymers with the drug, again indicating the crystalline nature of the drug in the mixture. Pure ARTM shows characteristic peaks intensities which indicate its % crystallinity. As shown in Figure 9 major specific intensities of ARTM are observed at 59045, 7826, and 9872. In the case of melt extrudates from F1 to F9, the characteristic of these peaks of ARTM disappeared and percentage crystallinity also decreases variably. While in the melt extrudates from F10–F15 the intensity of ARTM characteristic peaks has decreased to acceptable amount. From the XRD studies of both fresh and aged SD formulations confirms the amorphous nature of ARTM with the polymers after HME (Figures 7, 8, and 9).

#### 6.3.3. FTIR Studies

**Figure 10:**
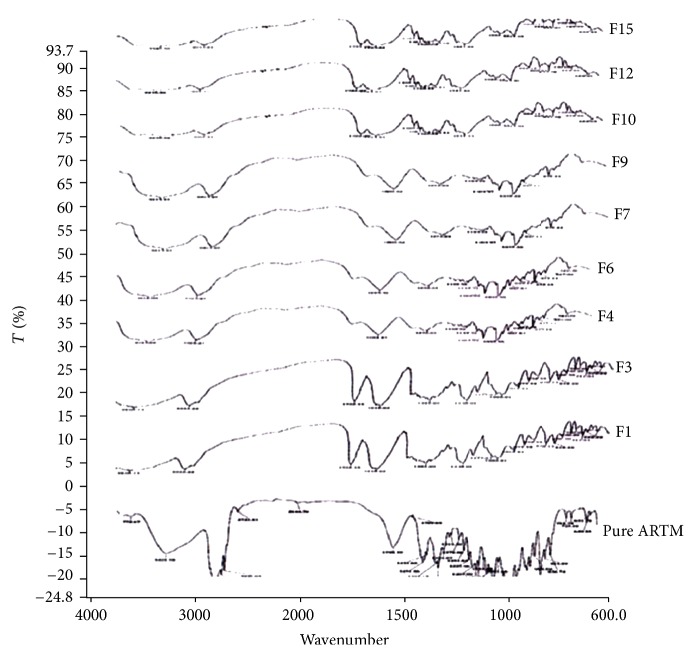
Infrared spectroscopic diagrams of pure drug and HME SD formulations.

Infrared spectroscopy has been widely used to investigate drug-polymer interactions in solid dispersion systems. In order to evaluate any possible chemical interactions between the drug and carriers, FTIR spectra of ARTM, physical mixtures, and HME formulations were examined (Figure 10). IR spectrum of ARTM presented characteristic peaks alkene at 3228–3420 cm^−1^ and OH stretch in the range of 2799–2950 cm^−1^. It also exhibited CO stretch at 1750 cm^−1^, CH stretching at 2750–2850 cm^−1^, and C–C stretch in the range of 1580–1650 cm^−1^. The formulations F1 and F3 showed characteristic peaks at 3414 cm^−1^, 2954 cm^−1^, 1604 cm^−1^, 1137 cm^−1^, and 991 cm^−1^. The formulations from F4 and F6 showed characteristic peaks at 3426 cm^−1^, 2936 cm^−1^, 1610 cm^−1^, 1199 cm^−1^, and 991.99 cm^−1^. The formulations from F7 and F9 showed characteristic peaks at 3424 cm^−1^, 2958 cm^−1^, 1608 cm^−1^, 1147 cm^−1^, and 991.87 cm^−1^. The formulations from F10, F12, and F15 showed characteristic peaks at range 3447 cm^−1^, 2961 cm^−1^, 1612 cm^−1^, 1144 cm^−1^, and 991.29 cm^−1^. The spectra of ARTM + Soluplus physical mixture and HME formulations are identical. The ARTM skeleton stretching vibrations are not affected by the addition of polymer, suggesting no interaction between the polymer and drug in the physical and HME mixtures. Lutrol has free hydrogen atoms that can potentially form hydrogen bonds with ARTM in the HME formulations. The carbonyl group is more favourable for hydrogen bonding and intermolecular interactions than the nitrogen atom because of steric hindrance. For HME formulations, the OH stretching bands broadened and the intensity of the bands decreased, indicating some degree of interaction between the proton donating groups of ARTM and the proton accepting groups in the Soluplus.

#### 6.3.4. SEM

**Figure 11:**
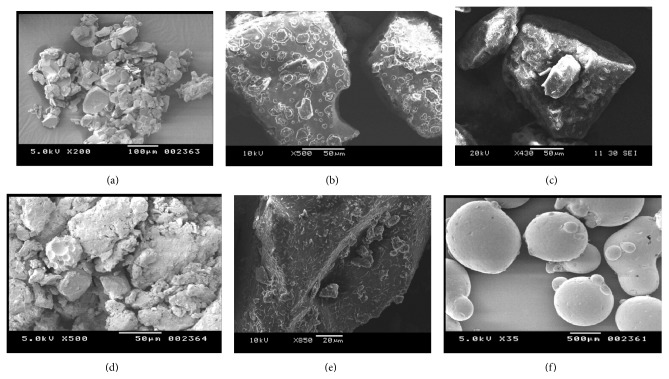
SEM images of (a) (pure ARTM), (b) (HME SD-F1), (c) (HME SD-F4), (d) (HME SD-F7), (e) (HME SD-F10), and (f) (Lutrol).

SEM micrographs of pure ARTM and ARTM HME SDs are shown in Figures 11(a)–11(e). From the SEM micrograph it was evident that HME of ARTM resulted in a significant particle size reduction of ARTM. SEM micrographs of pure ARTM revealed large crystalline blocks (Figure 11(a)), whereas ARTM SDs were found to be without sharp edges (b)–(e). The ARTM SDs appeared to be agglomerated with smooth surface owing to the presence of polymer.

#### 6.3.5. Dissolution Studies

**Figure 12:**
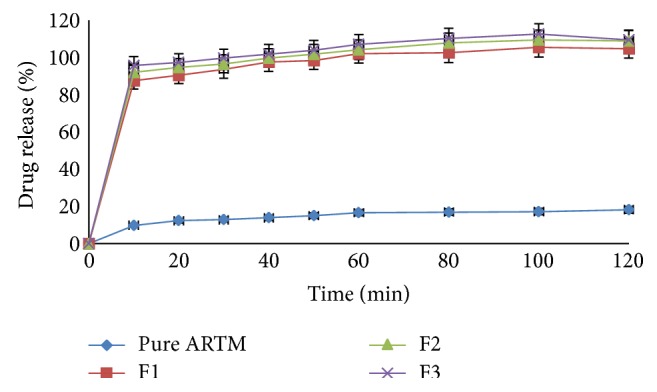
In vitro release of HME formulation batches F1 to F3 at phosphate buffer pH 7.2 with 1% SLS (mean ± SD (*n* = 3)).

**Figure 13:**
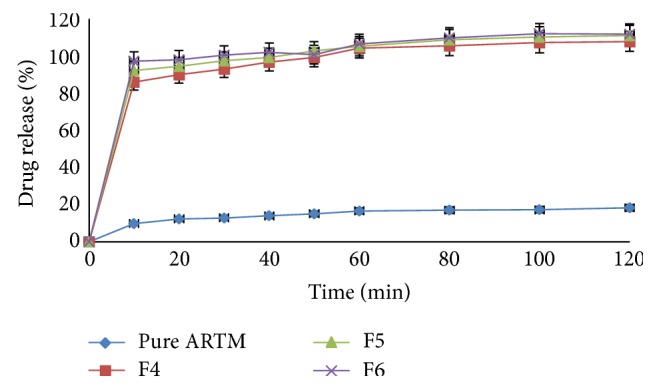
In vitro release of HME formulation batches F4 to F6 at phosphate buffer pH 7.2 with 1% SLS (mean ± SD (*n* = 3)).

**Figure 14:**
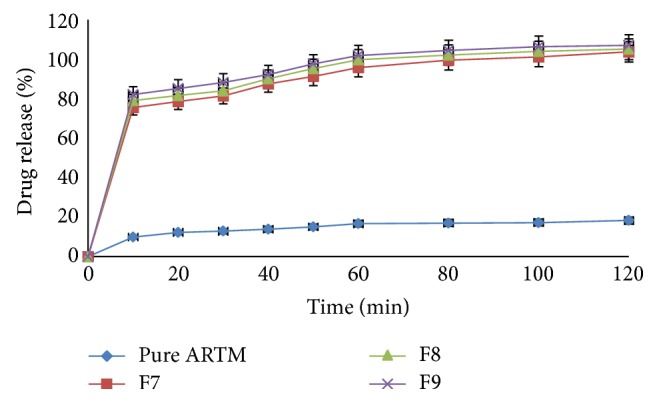
In vitro release of HME formulation batches F7 to F9 at phosphate buffer pH 7.2 with 1% SLS (mean ± SD (*n* = 3)).

**Figure 15:**
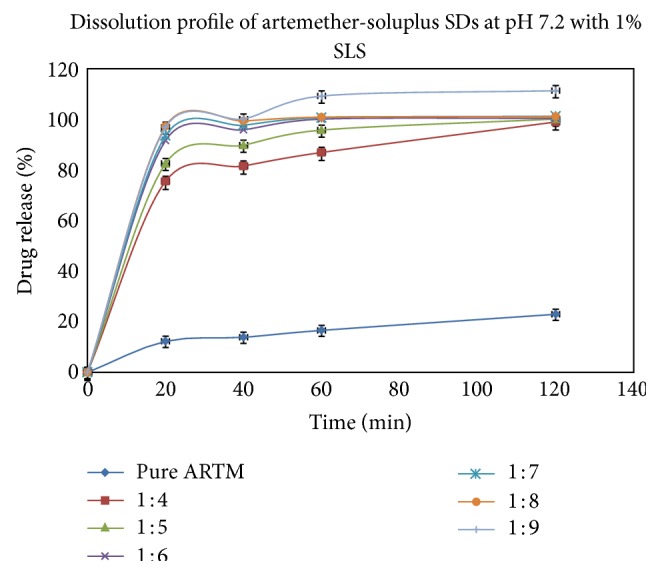
In vitro release of HME formulation batches F10 to F15 at phosphate buffer pH 7.2 with 1% SLS (mean ± SD (*n* = 3)).

**Figure 16:**
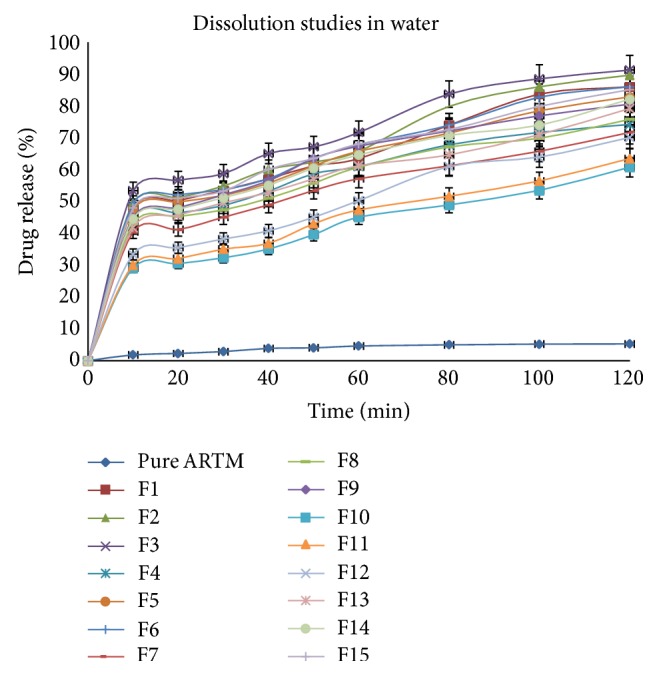
In vitro release of HME formulation batches F1 to F15 at distilled water with 1% SLS (mean ± SD (*n* = 3)).

Figures 12, 13, 14, and 15 show the dissolution profiles of various HME formulations and mixtures of ARTM with Soluplus-PEG 400, Soluplus-Lutrol F127, Soluplus-Lutrol F68, and Soluplus, respectively. Because of the extreme low solubility of the drug, 1% (w/v) SLS was added to the dissolution medium. ARTM is a poorly soluble drug with a solubility of 0.0183 *μ*g/mL in water. The saturation solubility of the ARTM was increased (be 0.17 *μ*g/mL) by the addition of SLS to the dissolution medium. The dissolution of the prepared HME formulations (F1 = 90.54%, F4 = 89.85%, F7 = 78.48%, F10 = 75.52%, at the end of  *T*  20 minutes) was approximately 7.37-, 7.32-, 6.39-, 6.15-fold higher than ARTM alone at the end of  *T*  20 minutes, respectively. The dissolution of the prepared HME formulations (F1 = 102.14%, F4 = 104.19%, F7 = 95.77%, F10 = 86.91%, at the end of  *T*  60 minutes) was approximately 6.15-, 6.27-, 5.76-, 5.23-fold higher than ARTM alone at the end of  *T*  60 minutes, respectively. The increase in the dissolution rate in the case of the HME formulation is attributed to the amorphous state of the drug that offers a lower thermodynamic barrier to dissolution and the formation of a glassy solution where the drug is molecularly dispersed in the polymer. The higher apparent solubility and increase in dissolution rate for amorphous materials are well known and have been extensively documented [24]. The enhancement in solubility is the result of the disordered structure of the amorphous solid. Because of the short-range intermolecular interactions in an amorphous system, no lattice energy has to be overcome, whereas in the crystalline material, the lattice has to be disrupted for the material to dissolve [25]. The solubility and dissolution rate of the drug were not enhanced by simple physical mixing with the polymer. Although SLS provided sufficient wetting of the drug particles as observed during dissolution studies, the hydrophilic polymer, Soluplus, in the physical mixture did not further enhance the dissolution of ARTM. The enhancement in dissolution in ARTM-Soluplus-PEG 400, ARTM-Soluplus-Lutrol F127 ARTM-Soluplus-Lutrol F68 and ARTM-Soluplus extrudates is also due to the conversion of crystalline drug into the amorphous state. The differences in the dissolution profile between these polymer systems are due to the solubility/dissolution nature of the polymer as well as surfactants in the dissolution medium. Dissolution of the drug in Soluplus alone is governed by the carrier, whereas in the case of Soluplus-surfactant systems, the dissolution rate is governed by solubilization of the polymer to create a hydrotropic environment for the insoluble drug. It was observed that in the dissolution studies the Soluplus-surfactant HME formulation dissolved rapidly, leaving the drug as a fine precipitate. The high-dissolution rate of ARTM from the Soluplus + PEG 400, Soluplus + Lutrol F127, and Soluplus + Lutrol F68 dispersion is believed to be due to the drug-polymer molecular intermixing at microlevel. The aqueous solubility and dissolution rate of prepared solid dispersion were significantly enhanced as shown in Figure 16.

#### 6.3.6. Effect of Lutrol F Grades [26, 27]

Lutrol F127 and Lutrol F68 (poloxamers) exist individually as monomolecular micelles. When the concentration of poloxamers in the system increases, this results in the formation of multimolecular aggregates. Polypropylene oxide (PPO) usually forms central hydrophobic cores, wherein methyl groups interact via Van der Wall's forces with the substance undergoing solubilization. However, water solubility is believed to be due to the polyethylene oxide (PEO) block by hydrogen bonding interactions of ether oxygen with water molecules. Because of these interactions, poloxamers are readily soluble in polar as well as nonpolar solvent medium.

#### 6.3.7. Stability on Storage [28]

Glassy solid dispersions are thermodynamically metastable systems that favour the conversion of amorphous form into the crystalline form under storage. To evaluate the physical state of the drug, the formulations were characterized by XRD and DSC after storage for 6 months. The formulations were stable during 6-month period. The dissolution stability was also evaluated for both initial and aged samples. As shown in the DSC thermograms in Figures 4 and 5, both HME formulations after storage were similar to the initial formulations and did not show any melting endotherm. This indicated an amorphous state of the drug in the aged samples. The XRD results as shown in Figures 7 and 8 demonstrate similar diffractograms of aged as compared with fresh HME formulations, indicating the amorphous nature of the ARTM. Both DSC and XRD results on aged samples confirmed that there was no recrystallization of the amorphous drug in the HME formulations, suggesting good physical stability. The dissolution profiles of aged samples found similar to those of fresh HME formulations further proved that the amorphous state of the drug was maintained in the aged formulations. The enhanced physical stability of the HME formulations upon storage is attributed to drug-polymer interactions and antiplasticization effect of the polymer. Soluplus-surfactant systems had strong intermolecular interactions, particularly hydrogen bonding between amorphous ARTM and the polymer [29]. These might further reduce the molecular mobility and retarded recrystallization during storage. The prepared SD were kept for stability studies at 37°C at room temperature and 40°C/70 RH (relative humidity). Samples were withdrawn at 3, 6 months and analysed for drug content. Percentage drug content was in the range of 97.37 ± 0.81% to 99.21 ± 0.48% in different ARTM formulations. All determinations are mean ± SD (*n* = 3).

#### 6.3.8. Mechanism of Dissolution [30]

**Figure 17:**
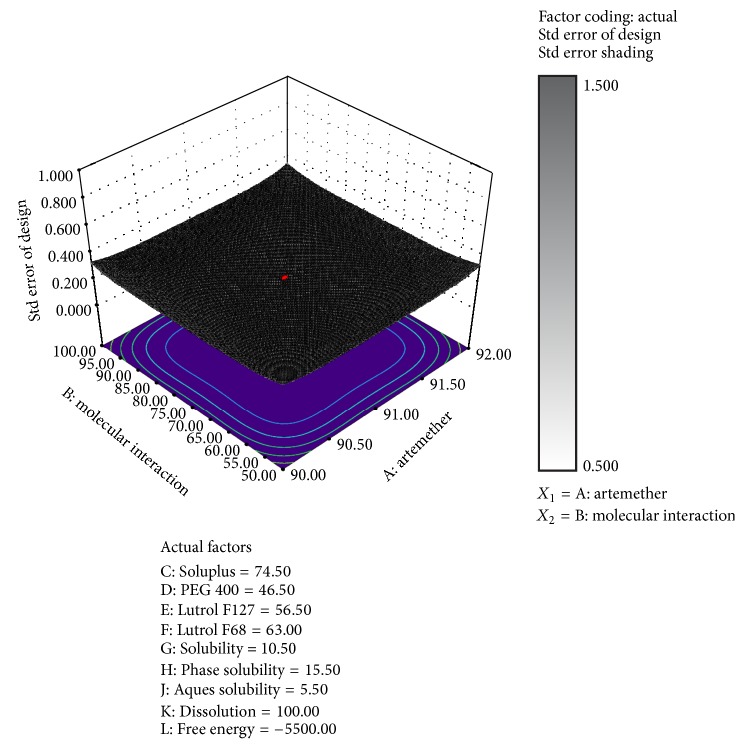
Drug-excipients molecular interaction studies using design expert software.

**Figure 18:**
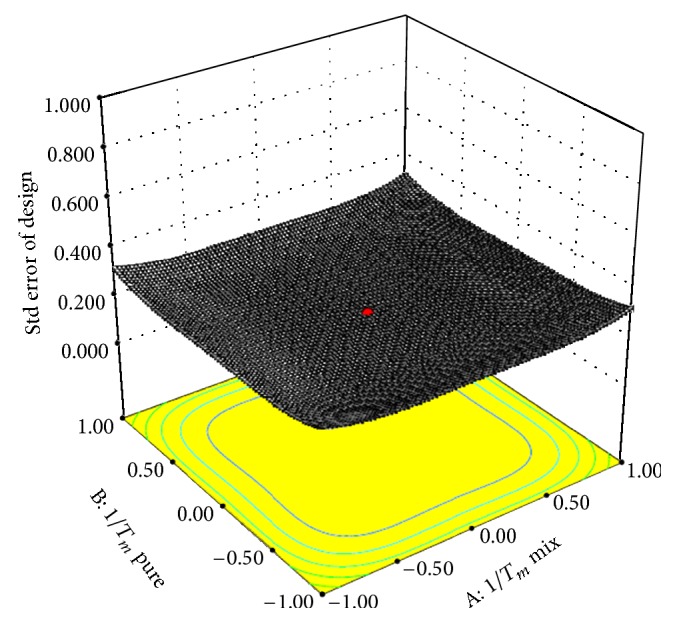
Contour surface plot of Florey-Huggins modelling in prepared SD formulations.

The dissolution kinetic studies were carried out and the best suited results were obtained in the case of Higuchi equation model. The value of *R* ^2^ in Higuchi model is nearer to 1.0 and thus we conclude that dissolution followed Higuchi order kinetics in Table 2. The drug excipient compatibility studies were carried out in which ARTM and molecular interaction were set as independent variables and others as dependent variables using Design Expert 8.0.7.1 trial version software shown in Figure 17. The results explain proactive interaction between the different excipient and ARTM as well as performance of parameters like solubility, phase solubility, free energy, and dissolution rate.

## 7. Results of Moisture Uptake Studies

Moisture uptake study is conducted to check hygroscopic nature of the prepared SD. No significant change in weight was observed after subjecting the sample to accelerated conditions of temperature and humidity. The accelerated stability studies showed that there was no considerable change in drug content during study duration. After moisture analysis of SD from F1 to F15 drug content was found to be same as compared to pure ARTM (99%).

### 7.1. Powder Flow Characterisation

The flow properties: bulk density and tapped density were carried out using standard procedures. Characteristic flow parameters like Angle of repose, Hausner's ratio, and Carr's index were calculated and results were indicated in Table 8. Results indicate that all the formulations have good flow properties in terms of the previously mentioned parameters.

### 7.2. In Vitro Antimalarial Activity

In vitro antimalarial activity showed that ARTM SD formulations from F1 to F15 were active against *P. falciparum* 3D7 at a very low concentration, Table 9. The IC_50_ value of ARTM SD powder was found to be in the range of 0.054 to 0.081 ng/mL. The IC_50_ value of HME formulations was 39 times lower than the IC_50_ value of pure ARTM (2.1 ng/mL) and 70 times lower than the IC_50_ value of standard antimalarial drug, chloroquine (3.8 ng/mL).

## 8. Conclusion

Dissolution rate and solubility enhancement of ARTM were obtained by preparing amorphous glassy solid dispersions using Soluplus, PEG 400, Lutrol F127, and Lutrol F68 polymers by hot-melt extrusion. The crystalline ARTM was converted to the amorphous state during the extrusion process with combined mixture of polymer and surfactants. DSC, XRD, IR data confirms that ARTM was converted to stable amorphous form using HME technology. Scanning microscopic analysis reveals smooth surface morphology as well as molecular interaction in prepared HME SD. Enhanced physical stability of the prepared HME formulations is attributed to drug-polymer interactions. HME formulations are less susceptible to recrystallization, perhaps due to the solubilising effect of the Soluplus. In vitro antimalarial studies showed enhanced activity for HME SD formulations as compared to that of standard drug chloroquine and ARTM. The improvement in the dissolution rate is in order of ARTM with Soluplus-Lutrol F127 > Soluplus-PEG 400 > Soluplus-Lutrol F68 > Soluplus only. The study revealed the importance of suitable carrier and processing technique selection, which can eventually enhance bioavailability of poorly soluble drug.

## Figures and Tables

**Table 1:** Comparative evaluation of ARTM and its HME formulations in terms of aqueous solubility, solubility in dissolution medium.

Batch	Formulation type	Ratio	Extrusion temp. (°C)	Batch size (gm)	Solubility in water (*μ*g/mL)	Solubility in disso. medium (*μ*g/mL)
Drug	Pure ARTM	—	—	—	0.0183 ± 1.3	0.254 ± 1.34
F1	ARTM : SOL : PEG 400	1 : 1.8 : 0.2	82-83	30	88.52 ± 1.54	221.2 ± 1.54
F2	ARTM : SOL : PEG 400	1 : 2.8 : 0.2	82-83	30	94.78 ± 1.12	222.9 ± 1.08
F3	ARTM : SOL : PEG 400	1 : 3.8 : 0.2	82-83	40	98.14 ± 1.69	216.2 ± 1.85
F4	ARTM : SOL : LF127	1 : 1.8 : 0.2	79-80	25	74.95 ± 1.47	210.2 ± 1.15
F5	ARTM : SOL : LF127	1 : 2.8 : 0.2	79-80	30	79.84 ± 1.29	213.3 ± 1.41
F6	ARTM : SOL : LF127	1 : 3.8 : 0.2	79-80	30	86.25 ± 1.36	218.4 ± 1.52
F7	ARTM : SOL : LF68	1 : 1.8 : 0.2	76-77	40	70.25 ± 1.12	219.2 ± 1.71
F8	ARTM : SOL : LF68	1 : 2.8 : 0.2	76-77	30	76.54 ± 1.24	218.3 ± 1.16
F9	ARTM : SOL : LF68	1 : 3.8 : 0.2	76-77	30	85.15 ± 1.56	220.6 ± 1.92
F10	ARTM : SOL	1 : 4	83-84	20	78.98 ± 1.24	213.8 ± 1.39
F11	ARTM : SOL	1 : 5	83-84	25	87.33 ± 1.71	217.2 ± 1.34
F12	ARTM : SOL	1 : 6	83-84	30	98.23 ± 1.39	219.4 ± 1.2
F13	ARTM : SOL	1 : 7	82-83	40	115.8 ± 1.45	222.8 ± 1.67
F14	ARTM : SOL	1 : 8	82-83	40	131.9 ± 1.85	224.3 ± 1.88
F15	ARTM : SOL	1 : 9	82-83	40	137.2 ± 1.05	231.7 ± 1.54

Artemether: ARTM, Soluplus: SOL, Polyethylene Glycol 400: PEG 400, Lutrol F127: LF127, and Lutrol F68: LF68 (mean ± SD (*n* = 3)).

**Table 2:** Order of drug release of various formulations determined by the regression coefficients.

Formulation types	Zero order	First order	Higuchi	Hixon Crowell	Krosmeyer Peppas
	(*r* ^2^)	(*r* ^2^)	(*r* ^2^)	(*r* ^2^)	(*r* ^2^)
F1	0.6792	0.2568	0.9976	0.7190	0.2289
F2	0.5726	0.2415	0.9979	0.7351	0.3656
F3	0.5573	0.3475	0.9980	0.7440	0.3860
F4	0.5814	0.3512	0.9973	0.7378	0.1193
F5	0.5664	0.3529	0.9980	0.7449	0.2946
F6	0.5664	0.3531	0.9986	0.6740	0.5579
F7	0.6371	0.4142	0.9952	0.7007	0.8875
F8	0.6192	0.4152	0.9955	0.8026	0.7095
F9	0.6098	0.4587	0.9962	0.7500	0.5500
F10	0.6630	0.2154	0.9940	0.6945	0.9685
F11	0.6005	0.2167	0.9969	0.7964	0.9265
F12	0.5640	0.2254	0.9983	0.7950	0.6851
F13	0.5754	0.2298	0.9980	0.7879	0.4377
F14	0.5709	0.3697	0.9974	0.6631	0.4857
F15	0.5807	0.4589	0.9969	0.6703	0.9735

**Table 3:** ARTM-Sol-PEG 400 formulations.

Conc. of polymer W/V	*S*_*s*_	*S*_0_	Δ*G*°tr
1	0.004713	0.000274	−7330.2
2	0.005995	0.000274	−7950.13
3	0.006491	0.000274	−8154.9
4	0.007285	0.000274	−8452.28
5	0.00832	0.000274	−8794.56

**Table 4:** ARTM-Sol-Lutrol F127 formulations.

Conc. of polymer W/V	*S*_*s*_	*S*_0_	Δ*G*°tr
1	0.003171	0.000274	−6309.41
2	0.003901	0.000274	−6842.83
3	0.004491	0.000274	−7205.83
4	0.005285	0.000274	−7625.34
5	0.00632	0.000274	−8086.15

**Table 5:** ARTM-Sol-Lutrol F68 formulations.

Conc. of polymer W/V	*S*_*s*_	*S*_0_	Δ*G*°tr
1	0.002713	0.000274	−5907.24
2	0.003008	0.000274	−6172.83
3	0.003949	0.000274	−6874.56
4	0.004285	0.000274	−7084.9
5	0.00532	0.000274	−7642.35

**Table 6:** ARTM-Sol formulations.

Conc. of polymer W/V	*S*_*s*_	*S*_0_	Δ*G*°tr
1	0.001713	0.000274	−4722.52
2	0.002008	0.000274	−5131.37
3	0.002491	0.000274	−5687.19
4	0.00285	0.000274	−6034.17
5	0.0032	0.000274	−6332.62

**Table 7:** Florey Huggins modelling of prepared formulations.

Ratio	1/*T*_*m*_mix	1/*T*_*m*_pure	LHS	*R*	Δ*H*_*f*_	*R*/Δ*H*_*f*_	ϕ_drug_	*m*	1/*m*	1 − 1/*m*	*ϕ*_polymer_	1 − 1/*m* ^*^ *ϕ*_polymer_	log(*ϕ*_drug_)	ln*ϕ*_drug_ + 1 − 1/*m* ^*^ *ϕ*_polymer_	*ϕ*_polymer_^2^	*R*/Δ*H*_*f*_(ln*ϕ*_drug_ + 1 − 1/*m* ^*^ *ϕ*_polymer_)	*R*/Δ*H*_f_^*^ *ϕ*_polymer_^2^ · *χ*	LHS	*χ*
F1	0.012	0.01	0.0011	8.314	61.842	−0.13	1	1	1	0	2	0	0	0	4	0	−0.537755772	0.001084011	0.001084
F2	0.012	0.01	0.0011	8.314	61.842	−0.13	1	2	0.5	0.5	3	1.5	0	1.5	9	−0.201658414	−1.209950487	0.001084011	−0.16558
F3	0.012	0.01	0.0011	8.314	61.842	−0.13	1	3	0.333333	0.6666667	4	2.666666667	0	2.666666667	16	−0.358503848	−2.151023088	0.001084011	−0.16558
F4	0.013	0.01	0.0014	8.314	61.842	−0.13	1	1	1	0	2	0	0	0	4	0	−0.537755772	0.001388889	0.001389
F5	0.013	0.01	0.0014	8.314	61.842	−0.13	1	2	0.5	0.5	3	1.5	0	1.5	9	−0.201658414	−1.209950487	0.001388889	−0.16528
F6	0.013	0.01	0.0014	8.314	61.842	−0.13	1	3	0.333333	0.6666667	4	2.666666667	0	2.666666667	16	−0.358503848	−2.151023088	0.001388889	−0.16528
F7	0.013	0.01	0.0019	8.314	61.842	−0.13	1	1	1	0	2	0	0	0	4	0	−0.537755772	0.001875902	0.001876
F8	0.013	0.01	0.002	8.314	61.842	−0.13	1	2	0.5	0.5	3	1.5	0	1.5	9	−0.201658414	−1.209950487	0.002046784	−0.16462
F9	0.013	0.01	0.002	8.314	61.842	−0.13	1	3	0.333333	0.6666667	4	2.666666667	0	2.666666667	16	−0.358503848	−2.151023088	0.002046784	−0.16462
F10	0.012	0.01	0.0008	8.314	61.842	−0.13	1	4	0.25	0.75	4	3	0	3	16	−0.403316829	−2.151023088	0.000793651	−0.18671
F11	0.012	0.01	0.0008	8.314	61.842	−0.13	1	5	0.2	0.8	5	4	0	4	25	−0.537755772	−3.360973575	0.000793651	−0.15921
F12	0.012	0.01	0.0012	8.314	61.842	−0.13	1	6	0.166667	0.8333333	6	5	0	5	36	−0.672194715	−4.839801948	0.001234568	−0.13765
F13	0.012	0.01	0.0008	8.314	61.842	−0.13	1	7	0.142857	0.8571429	7	6	0	6	49	−0.806633658	−6.587508206	0.000793651	−0.12166
F14	0.012	0.01	0.0011	8.314	61.842	−0.13	1	8	0.125	0.875	8	7	0	7	64	−0.941072601	−8.604092351	0.001084011	−0.10829
F15	0.013	0.01	0.0015	8.314	61.842	−0.13	1	9	0.111111	0.8888889	9	8	0	8	81	−1.075511544	−10.88955438	0.001547117	−0.09722

**Table 8:** Representation of the powder flow characteristics of various formulations.

Formulation	(Angle of repose *θ*)	Bulk density	Tapped density	Hausner's ratio	Carr's index
F1	20.61	0.3586	0.4428	1.23	19.01
F2	20.54	0.3581	0.4416	1.23	18.91
F3	21.14	0.3612	0.4366	1.2	17.26
F4	20.96	0.3508	0.4025	1.14	12.84
F5	20.1	0.3434	0.3996	1.16	14.07
F6	20.61	0.3484	0.4035	1.15	13.65
F7	21.48	0.3503	0.4016	1.14	12.75
F8	18.74	0.3516	0.4048	1.15	13.15
F9	19.23	0.3403	0.3852	1.13	11.64
F10	20.27	0.3852	0.4752	1.23	18.95
F11	21.23	0.3861	0.4739	1.22	18.53
F12	18.49	0.3776	0.4444	1.17	15.03
F13	18.39	0.3663	0.4428	1.2	17.28
F14	17	0.3367	0.3987	1.18	15.55
F15	16.32	0.3419	0.3952	1.15	13.47

**Table 9:** In vitro antimalarial activity of various formulations.

Formulation codes	IC_50_ (ng/mL) (in vitro whole cell SYBR assay study)	Chloroquine-sensitive cell line (ng/mL) 3D7
F1	0.062	0.0000089
F2	0.059	0.0000078
F3	0.054	0.0000067
F4	0.069	0.0000098
F5	0.064	0.0000088
F6	0.059	0.0000076
F7	0.074	0.0000099
F8	0.071	0.0000094
F9	0.068	0.0000081
F10	0.081	0.0000095
F11	0.078	0.0000085
F12	0.074	0.0000065
F13	0.065	0.0000058
F14	0.058	0.0000039
F15	0.056	0.0000028
ARTM	2.1	0.00068
Chloroquine	3.8	0.0025
